# Highly Cancellous Titanium Alloy (TiAl_6_V_4_) Surfaces on Three-Dimensionally Printed, Custom-Made Intercalary Tibia Prostheses: Promising Short- to Intermediate-Term Results

**DOI:** 10.3390/jpm11050351

**Published:** 2021-04-28

**Authors:** Wiebke K. Guder, Jendrik Hardes, Markus Nottrott, Lars E. Podleska, Arne Streitbürger

**Affiliations:** Department of Orthopedic Oncology, University Hospital Essen, Hufelandstrasse 55, 45147 Essen, Germany; jendrik.hardes@uk-essen.de (J.H.); markus.nottrott@uk-essen.de (M.N.); lars-eric.podleska@uk-essen.de (L.E.P.); arne.streitbuerger@uk-essen.de (A.S.)

**Keywords:** highly cancellous, implant surface, tibia, titanium alloy, 3D printing, megaendoprosthesis, orthopedic oncology

## Abstract

Custom-made, three-dimensionally-printed (3D) bone prostheses gain increasing importance in the reconstruction of bone defects after musculoskeletal tumor resections. They may allow preservation of little remaining bone stock and ensure joint or limb salvage. However, we believe that by constructing anatomy-imitating implants with highly cancellous titanium alloy (TiAl_6_V_4_) surfaces using 3D printing technology, further benefits such as functional enhancement and reduction of complications may be achieved. We present a case series of four patients reconstructed using custom-made, 3D-printed intercalary monobloc tibia prostheses treated between 2016 and 2020. The mean patient age at operation was 30 years. Tumor resections were performed for Ewing sarcoma (*n* = 2), high-grade undifferentiated pleomorphic bone sarcoma (*n* = 1) and adamantinoma (*n* = 1). Mean resection length was 17.5 cm and mean operation time 147 min. All patients achieved full weight-bearing and limb salvage at a mean follow-up of 21.25 months. One patient developed a non-union at the proximal bone-implant interface. Alteration of implant design prevented non-union in later patients. Mean MSTS and TESS scores were 23.5 and 88. 3D-printed, custom-made intercalary tibia prostheses achieved joint and limb salvage in this case series despite high, published complication rates for biological and endoprosthetic reconstructions of the diaphyseal and distal tibia. Ingrowth of soft tissues into the highly cancellous implant surface structure reduces dead space, enhances function, and appears promising in reducing complication rates.

## 1. Introduction

Personalized, custom-made implants have gained importance in the reconstruction of bone defects after musculoskeletal tumor resections. Megaendoprostheses are a well-established and accepted reconstruction technique of osteoarticular defects of the hip, knee, and glenohumeral joint [1]. However, depending on the amount of remaining bone stock and soft tissue coverage, standard megaendoprosthetic implants are either unavailable or associated with higher complication rates in more distally located sites such as the distal tibia and ankle [2,3,4,5,6,7]. Since three-dimensional (3D) computer-assisted design (3D-CAD) and 3D-printing technology were introduced in the production process of orthopedic implants, the availability of patient-individualized stems and anatomy-imitating implants in complex anatomic and biomechanical sites has improved the rates of joint and limb salvage of both osteoarticular and intercalary reconstructions [8,9,10].

3D printing technology also has its implications in improving osseointegration by creating implant designs with highly cancellous surfaces [11,12].

To our knowledge, the ingrowth of soft tissues into highly cancellous implant surfaces as a means of reducing dead space around megaendoprostheses, improving periprosthetic infection rates and enhancing functional outcome remains largely unexplored. Soft tissue attachment to titanium implants has only been investigated in the context of intraosseous transcutaneous amputation prostheses to serve as a barrier for exogenous agents such as bacteria [13].

For this reason, we present the short- to intermediate-term results of a case series of four patients treated by intercalary tumor resection for tumors of the distal tibia and reconstruction using a novel, custom-made 3D-printed monobloc implant design and a highly porous surface. The presented implant design avoids distal tibia resection—in favor of intercalary resection—in all cases despite little remaining bone stock above the ankle joint.

## 2. Materials and Methods

Four patients, who underwent tibial intercalary resection for malignant primary bone tumors, were reconstructed using 3D-printed intercalary monobloc tibia megaendoprostheses with highly cancellous titanium alloy surfaces (TiAl_6_V_4_) between 2016 and 2020.

Patient data were prospectively collected and retrospectively analyzed.

### 2.1. Patient Characteristics

The mean patient age at the time of operation was 30 years (range 19–54 years). In all cases, the diagnosis of a malignant primary bone tumor was confirmed by incisional biopsy. Histological diagnoses in decreasing order were Ewing sarcoma (*n* = 2), high-grade undifferentiated pleomorphic bone sarcoma (UPS), and adamantinoma in one case each. Three patients received neoadjuvant and adjuvant chemotherapy. None of the patients received (neo-)adjuvant radiation treatments of the primary tumor site. Comorbidities were absent in all treated patients.

Three patients underwent tumor resection and reconstruction using an intercalary tibia monobloc implant within the same operation, one patient was temporarily reconstructed using a polymethyl methacrylate (PMMA) spacer and underwent definite reconstruction after completion of chemotherapy. The mean tumor resection and reconstruction length was 17.5 cm with a mean of 28.75 mm remaining distal tibia. Bone growth stimulants or postoperative drugs to enhance tissue growth were not administered. The mean operation time was 147 min. All monobloc prostheses were implanted in a non-cemented fashion. Local muscle flaps were performed for adequate soft tissue coverage of the implants. All patients ambulated with partial weight-bearing of 20 kg using crutches for 6 weeks after the operation. Weight-bearing was then increased at increments of 10 kg on a weekly basis.

All four patients were alive at the time of retrospective analysis without evidence of disease at a mean follow-up of 21.25 months (range 5–52 months).

Patient and operation-specific data are detailed in Table 1.

### 2.2. Indication

To be eligible for reconstruction using a custom-made 3D printed intercalary titanium alloy implant with a highly cancellous surface, patients had to meet the following criteria:

- Primary malignant bone tumor of the diaphysis and metaphysis of the distal tibia;
- Remaining bone stock in the distal tibia excluded use of off-the-shelf intercalary tibial megaendoprosthetic implants and stems;
- Absence of comorbidities affecting bone and wound healing, such as diabetes mellitus, peripheral artery occlusive disease, or positive smoking history;
- Patient consent to undergo this reconstruction rather than below-knee amputation or other biological reconstruction.

### 2.3. Pre-Operative Planning and Production

**Figure 1:**
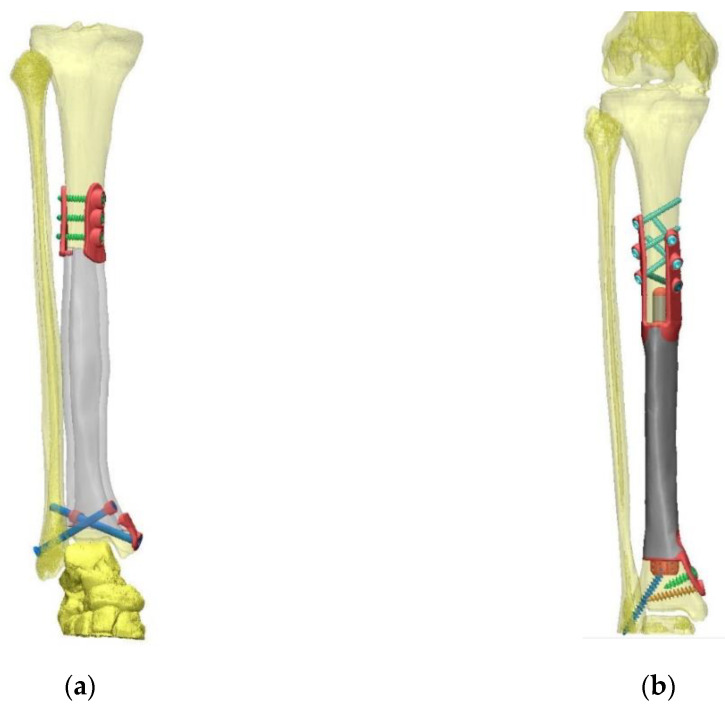
3D-CADs based on computed tomography datasets (**a**) Patient #1: hollow implant with a highly cancellous implant surface and extracortical plates with supplementary interlocking screw options at proximal and distal implant-bone interface; (**b**) Patient #4: solid implant with a highly cancellous implant surface, small solid proximal and hollow distal stem and plates with interlocking screw options at the proximal and distal implant-bone interface.

Custom-made 3D-printed intercalary tibia monobloc titanium aluminum vanadium alloy (TiAl_6_V_4_) implants with highly cancellous implant surfaces were planned on preoperative computed tomography (CT) scans (DICOM format, reconstruction matrix 512 × 512, slice thickness ≤ 1 mm). Osteotomy levels were defined using corresponding pre-treatment magnetic resonance imaging (MRI) studies and measured by distance to the adjacent joint line. 3D-CAD of the implants was performed by Implantcast Inc. (Buxtehude, Germany) as specified and approved by the operating surgeon before production using electron beam melting technology (EBM^®^) commenced (Figure 1). Implants were gamma-sterilized using the same parameters established for off-the-shelf implants.

### 2.4. Implant Properties and Highly Cancellous Implant Surface (EPORE^®^, Implantcast, Buxtehude, Germany)

All custom-made 3D-printed intercalary tibia monobloc titanium alloy (TiAl_6_V_4_) implants were designed imitating the individual patient’s bone geometry and dimensions (Table 2).

**Figure 2:**
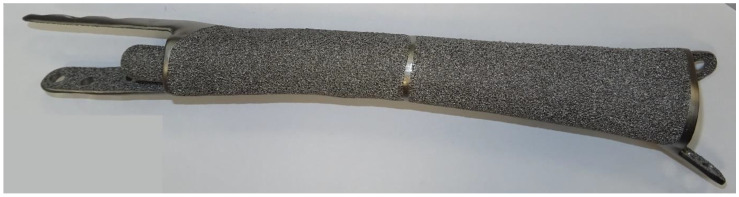
Photograph of a finished monobloc solid body implant (Patient #2) with highly cancellous implant surface on stem, bone-facing extracortical plates, and implant body.

Implants were manufactured with a highly cancellous implant surface (EPORE^®^) characterized by trabeculae with a diameter of 330–390 µm to imitate trabecular bone and promote tissue ingrowth (Figure 2).

The implant was designed with a hollow prosthetic body for patient #1, while a solid body was used for following implants.

Stems or extracortical plates with supplementary interlocking screw options were used to anchor the implants to adjacent bone at implant-bone interfaces. At the proximal interface, solid stem designs were used; distally hollow stems were planned whenever feasible depending on remaining bone stock.

### 2.5. Surgical Technique

The stem length of tibial monobloc implants with a proximal and distal stem is a limiting factor for successful implantation. If the stem dimensions are chosen too long, they will pose an obstacle to repositioning the tibia. For this reason, the implant design of the monobloc implant of patient #4 included a distal stem with a length of only 10 mm. After resection of the distal diaphyseal tibia, axial, angular, and rotational maneuverability of the lower leg are increased even when an intact fibula remains. Therefore, in the case of patient #4, the proximal stem was implanted first while the remaining distal tibia and foot were lowered and rotated to the side as much as possible to prevent interference with the proximal implantation. When resetting the distal tibia with the distal implant interface and stem, the existing soft tissue expansibility was used to gain the leeway necessary for implanting the 10 mm distal stem. If implantation of the distal stem had proven impossible for a lack of leeway, an additional fibular osteotomy would have been performed to gain more clearance. As the maximum amount of contrivable clearance is limited, implantation of longer implant stems would need to be planned with a modular implant design.

### 2.6. Bone Ingrowth

Bone ingrowth at the implant-bone interfaces was assessed clinically and radiographically. An absence of pain or instability (after full weight-bearing was achieved) served as a clinical indicator for bone ingrowth. Radiographic criteria on postoperative plain radiographs included correct implant positioning without dislocation or signs for aseptic loosening. In the event of clinical or radiographic symptoms, additional CT imaging of the reconstruction was performed.

### 2.7. Complication Assessment

Complications were categorized according to the Henderson classification [14].

### 2.8. Functional Assessment

The Musculoskeletal Tumor Society (MSTS) score and Toronto Extremity Salvage Score (TESS) were used to assess functional outcomes [15,16]. The respective questionnaires were handed out in paper form and completed by patients as part of their outpatient follow-up examinations.

## 3. Results

**Figure 3:**
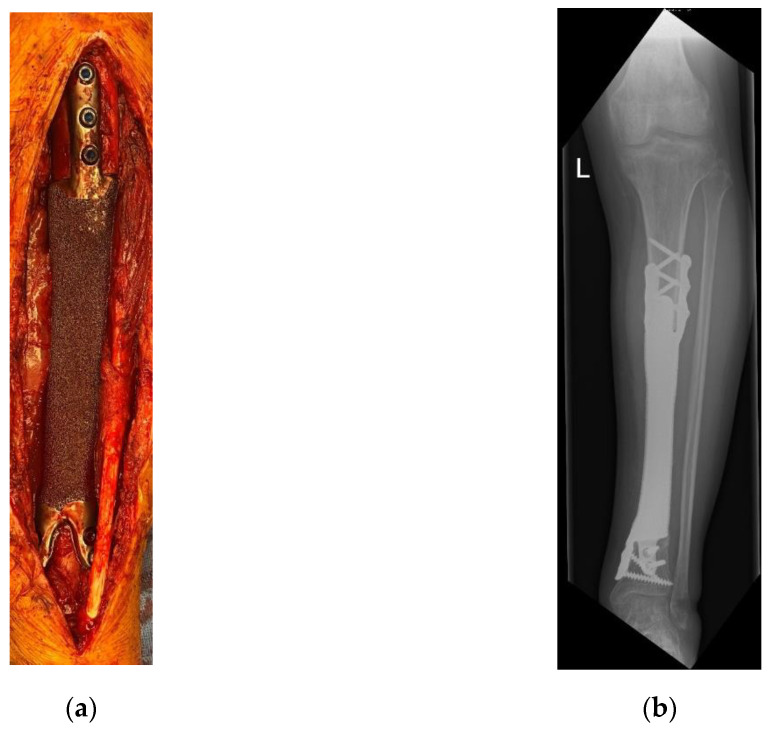
Patient #3: Reconstruction after implantation of a monobloc intercalary distal tibia implant: (**a**) intraoperative image; (**b**) postoperative radiograph of the tibia anterior-posterior (a.p.) view. L means left.

In the four patients—reconstructed using custom-made 3D-printed intercalary megaendoprostheses with a highly cancellous implant surface—distal tibia resection and below-knee amputation were avoided in all cases (Figure 3).

### 3.1. Bone Ingrowth

Primary ingrowth of the implant occurred in all patients at both implant-bone interfaces except for the proximal osteotomy line of patient #1. A partial non-union observed in this patient is more comprehensively analyzed in Section 3.3. At the current follow-up, we did not observe differences in osseointegration among treated patients (regardless of age at operation).

### 3.2. Soft Tissue Ingrowth

**Figure 4:**
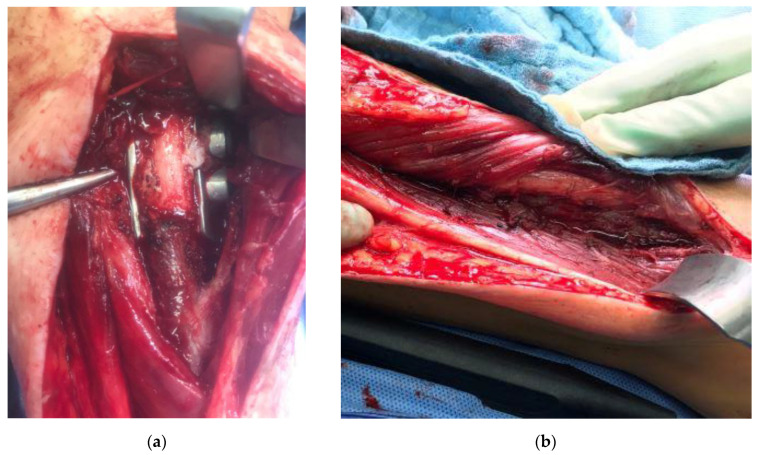
Patient #1: Intraoperative images during revision operation nine months after primary reconstruction: (**a**) view of proximal implant-tibia interface with visibility of partial non-union and soft tissue ingrowth into the highly cancellous implant surface; (**b**) complete ingrowth of muscles and soft tissues into the highly cancellous implant body surface.

A complete ingrowth of muscular tissue into the highly cancellous implant surface was confirmed in one patient who underwent a revision for partial non-union (Figure 4).

### 3.3. Complications

**Figure 5:**
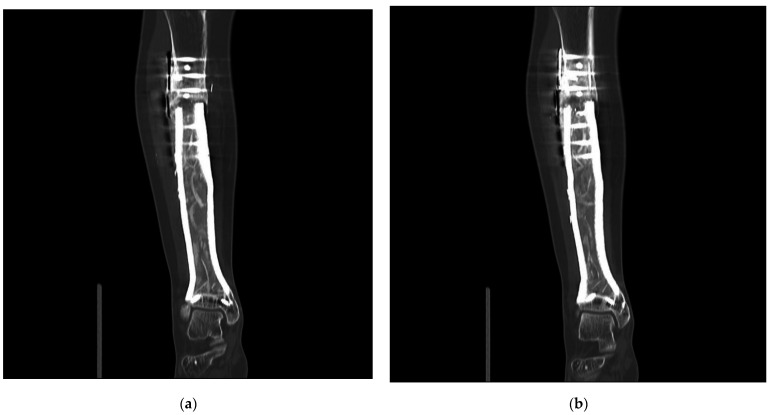
Patient #1: Computed tomography scan 43 months after primary reconstruction: (**a**,**b**) coronar view of the implant with the depiction of the bone graft-loaded hollow implant cavity and persistent non-union of the proximal implant-bone interface.

**Figure 6:**
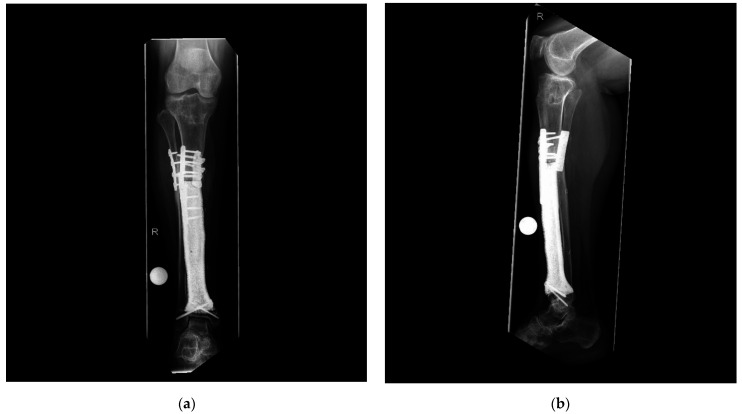
Patient #1: Postoperative radiographs after osteosynthetic plating of the proximal implant-bone interface (**a**) a.p. view; (**b**) lateral view. R means right.

The first patient reconstructed using a highly cancellous 3D-printed monobloc intercalary tibia implant developed an incomplete non-union at the proximal bone-implant interface (Henderson Type 3—structural failure). Two extracortical plates with supplementary interlocking screws bridging the bone-implant interface were used to anchor the implant to the proximal tibial diaphysis without a central stem. This anchorage design was chosen to allow filling the hollow implant body with autologous iliac crest graft (Figure 5). Operative revision and additional plating of the bone implant interface while retaining the original implant were performed 9 months after primary reconstruction (Figure 6). In addition, the ipsilateral fibula was osteotomized and fixed to the tibial column using screw osteosyntheses after roughening the facing bone cortices to encourage bone union. A hypertrophic pseudarthrosis recurred at the tibial bone-implant interface while the fibular transfer consolidated and continues to stabilize the reconstruction by taking part of the load. The patient currently has full weight bearing using a light brace and declines further operative revision as her activities of daily life are not impaired and she has no athletic ambitions. Implant design has been adapted to include a central stem and solid implant body at the proximal bone-implant interface. After this alteration, non-union and hypertrophic pseudarthrosis were avoided in later patients.

Soft tissue failure (Henderson type 1), aseptic loosening (Henderson type 2), periprosthetic infections (Henderson type 4), or local recurrence (Henderson type 5) were not observed in this collective.

### 3.4. Functional Outcome

All patients have achieved full weight-bearing and returned to their activities of daily life. The mean MSTS and TESS scores were 23.5 and 88, respectively. Patient #4 completed the functional questionnaire three months after the operation when presenting at the outpatient clinic for her first postoperative follow-up. She has not completed a functional rehabilitation program due to ongoing adjuvant chemotherapy yet.

## 4. Discussion

In this study, we present the short- to intermediate-term outcomes of four patients reconstructed using 3D-printed patient-individualized intercalary tibia monobloc prostheses with highly cancellous implant surfaces. From our point of view, complete ingrowth of soft tissues into the highly cancellous implant surface and continued joint salvage of the ankle joint despite little remaining bone stock are the most significant findings of this study.

The rationale for using titanium aluminum vanadium alloy (TiAl_6_V_4_) implants for the presented implant design were based on two main considerations: biocompability and choice of available production technique. Titanium alloys are a certified and reliable material with good biocompability for non-cemented reconstructions. They also have sufficient stability and processed using EBM allows for highly porous implant surfaces.

Periprosthetic infection is a serious problem affecting primary and revision total joint arthroplasties [17,18], but infection rates of megaendoprosthetic reconstructions are even higher despite implant features such as silver coating [19]. Possible reasons are larger reconstruction lengths with larger implant surfaces, longer operation times, and frequently immunocompromised patients. McConoughey et al., report that bacteria are often introduced into the wound in their planktonic form, growing in joint fluids before colonizing an implant. Later, they form biofilms to avoid exposure to high doses of antibiotics, develop resistance, and persist in a dormant state [20].

Complete ingrowth of soft tissues into the implant surface, as observed in this study, addresses many known causes and promotive factors in the development of periprosthetic infection: reduction of (a) dead space, (b) scar tissue formation surrounding the implants at a distance, and (c) excessive joint fluid formation around the implant.

In a study by Cordero et al., rough titanium alloy surfaces have shown a higher tendency of bacterial colonization when compared with smooth surfaces [20,21]. However, soft tissue ingrowth and accessibility of implant surfaces for immune cells may balance this observed disadvantage. While we concede that a lack of periprosthetic infection in the presented case series is not sufficient to make any firm conclusions about the impact of soft tissue ingrowth on implant surfaces, highly cancellous implant surfaces seem a feasible implant modification warranting further research.

Bone and soft tissue ingrowth also have implications for functional outcomes. MSTS and TESS scores of 23.5 and 88 in this study were satisfactory and most likely caused by the preservation of the ankle joint as well as soft tissue ingrowth. They compete with or exceed functional outcomes observed after biological and endoprosthetic intercalary or osteoarticular distal tibia reconstructions. Tanaka et al. reported MSTS scores of nineteen patients ranging between 93–100% after reconstruction with vascularized fibula grafts in intercalary femur and tibia defects. They also observed a union rate of 79% after a mean time of 7.8 months, which necessitated long periods of partial to no weight-bearing [22]. Khira et al., published a mean MSTS score of 84 (80–92) in their collective of patients reconstructed using vascularized fibula grafts with an Ilizarov external fixator for large tibial bone defects [23]. Intercalary or osteoarticular distal tibia allografts (optionally augmented with vascularized fibula grafts or composite prostheses) are another biological reconstruction option reported by Donati et al. However, they were rarely indicated for the ankle joint compared with other locations (*n* = 3). Complications included non-union (49%) and fracture (27%) observed in all reconstructed sites (*n* = 112) [24]. 

Abudu et al. reported their results for endoprosthetic replacement of the distal tibia and ankle joint (*n* = 5). While function was excellent to begin with, it deteriorated over time. Yet, patients maintained a mean Enneking score of 50% in this study [5]. In 2017, Yang et al. documented a median MSTS score of 66% in eight patients treated by custom-made distal tibia megaprosthesis [2]. Lee et al. assessed a mean MSTS score of 24.2 in six patients treated with the custom-made, hinged distal tibia and ankle prostheses. Among complications, talar collapse and wound infection were noted [6]. Shekkeris et al. reported that two of six patients treated by endoprosthetic distal tibia replacement went on to have below-knee amputation for persistent infection after a mean of 16 months in their study. The mean MSTS and TESS score of patients retaining the implants was 70% and 71% [3]. The most common complication after endoprosthetic intercalary reconstruction observed by Streitbürger et al. was aseptic loosening. Alteration of stem design to fit biomechanical demands of epi- or metaphyseal stem anchorage showed a tendency to improve implant longevity, though. [10].

The complication rates presented in the above-mentioned studies prove that reconstruction of bone defects of the distal tibia after tumor resections remains challenging regardless of the reconstruction technique chosen. Furthermore, the authors agree that below-knee amputation remains a valid treatment choice. The implant design presented in this case series achieved joint and limb salvage at a low complication rate and satisfactory functional outcomes with an early return to full weight-bearing. For this reason, increased consideration of biomechanical demands on implants and further technological advancements of 3D-printing seem a promising research avenue to increase the role of megaendoprosthetic reconstructions in this challenging location.

## 5. Conclusions

Reconstruction of the diaphyseal and distal tibia using custom-made 3D-printed intercalary implants proves to be a feasible treatment strategy in this case series. Considering that functional outcome after below-knee amputation leads to acceptable results and lower complication rates compared with other limb salvaging biological and standard megaendoprosthetic approaches, a low complication rate and good functional outcome in this case series emphasize that limb salvage using 3D-printed custom-made implants should be considered when counseling patients despite a lack of long-term experiences. However, observation of these patients with regard to long-term functional results and complication rates is necessary. Highly cancellous implant body designs should be considered in other megaendoprosthetic implant sites as well and future studies investigating this design’s advantages and complications seem warranted.

## Figures and Tables

**Table 1:** Patient and operation-specific characteristics.

#	1	2	3	4
Age (years)	25	19	22	54
Diagnosis	Adamantinoma	Ewing	Ewing	UPS
Grading	low-grade	high-grade	high-grade	high-grade
Resection length (mm)	175	200	160	165
Remaining distal tibia (mm)	5	30	45	35
Operation time (minutes)	NA	210	125	106
Margin (R)	0	0	0	0
Chemotherapy	-	+	+	+
Radiation	-	-	-	-
Complication	Non-union (proximal)	-	-	-
MSTS	20	29	25	20
TESS	86	100	96	70
Follow-up (months)	52	18	10	5

# number.

**Table 2:** Mechanical Implant Properties.

#	1	2	3	4
Reconstruction Length (mm)	175	200	160	165
Implant body	hollow	solid	solid	solid
Proximal stem (mm)	none	Solid 14 × 20	solid 14 × 28	solid 11 × 25
Proximal extracortical plates (mm)				
medial	41	55	60	67
lateral	38	65	55	76
Distal stem (mm)	none	none	none	hollow 20 × 10
Distal extracortical plates (mm)				
medial	12	25	38	28
ventral	-	18	28	-

# number.

## Data Availability

The data presented in this study are available on reasonable request from the corresponding author.
